# Achievements of Diabetes Goals and Their Determinants in Type 2 Diabetic Patients Attending Outpatient Diabetic Clinic in Northern Ethiopia

**DOI:** 10.1155/2017/5713187

**Published:** 2017-12-31

**Authors:** Ezra Belay, Abel Abera, Aman Mehari, Gidey Gebremeskel, Asrat Endrias, Kedir Endris

**Affiliations:** ^1^Department of Medical Biochemistry, College of Health Sciences, Mekelle University, 1871 Mekelle, Ethiopia; ^2^Department of Nursing, College of Health Sciences, Mekelle University, 1871 Mekelle, Ethiopia

## Abstract

**Background:**

The aim of this study was to assess target diabetic goal achievements and to explore variables associated with them.

**Methods:**

A cross-sectional study was conducted between December 2015 and April 2016 on 188 type 2 diabetic patients attending Ayder Referral Hospital's outpatient diabetic clinic. Glycemic control was assessed using fasting plasma glucose values and total cholesterol and triglyceride were used to evaluate lipid profiles. Bivariate and multivariate logistic regression analyses were done to identify factors associated with poor glycemic control, hypertension, and dyslipidemia.

**Result:**

Mean duration of diabetes was 6.5 years. Combined glycemic, lipid, and blood pressure targets were achieved only in 8.5% of the participants. More males achieved combined targets than females. Separately, while above two-thirds of the patients had poor glycemic control (67%), more than half of the participants have had poor lipid (58.5%) and blood pressure (52.1%) control. A significant portion of the patients (68.1%) had also comorbidities other than hyperglycemia. In bivariate and multivariate analyses, longer duration of diabetes disease (AOR: 3.4; *P* = 0.013) and marked month to month fasting plasma glucose (FPG) variability as measured by large standard deviation (AOR: 2.5; *P* = 0.023) were significantly associated with overall poor mean FPG results. Female sex was also significantly associated with dyslipidemia (AOR: 1.9; *P* = 0.049).

**Conclusion:**

The study showed that achievements of combined diabetic goals are generally poor.

## 1. Introduction

More than 422 million (8.5%) adult people were living with diabetes at the end of 2014. Tens of millions suffer disabling and life-threatening complications of diabetes such as heart attack, stroke, kidney failure, blindness, and amputation [1, 2]. The proportion of premature deaths due to diabetes is higher in developing countries than in developed countries and the prevalence is rising most rapidly in these least developed countries [2]. According to 2014 International Diabetes Federation (IDF) report, more than 22 million diabetic people (about 7.1%) were living in Africa [3]. Diabetes is becoming a growing health problem along with other noncommunicable diseases in Ethiopia. Cross-sectional studies estimated the prevalence of diabetes to be as high as 7% and 5% of deaths are due to diabetes mellitus [4, 5].

Optimal control of plasma glucose, lipid profile, and blood pressure is central to the management of diabetes mellitus. These are the key factors in reducing morbidity and death from the disease. Several large studies have proven that achieving these diabetic goals averts or considerably delays life-threatening complications of diabetes, enabling people with diabetes to live longer and healthier lives [6, 7]. Despite the strong consensus that excellent management of diabetes prevents both macrovascular and microvascular outcomes in type 1 and type 2 diabetes mellitus, universal studies indicated that majority of diabetic patients did not attain their target diabetic goals [8, 9]. This is even more challenging in developing countries due to a limited access to adequate health services, poor education level, reduced access to medical education, and a lack of standard laboratory tests such as HbA1c for assessing metabolic controls. Moreover, besides the fact that low socioeconomic status is associated with higher prevalence of diabetes and a greater risk for diabetes complications, there are likely many specific elements of low economic status which predispose diabetic patients in resource-limited setting to poor diabetes control [10–12]. The aim of this study was to assess the extent of diabetic goal achievements and failures and their determinants in patients with diabetes in Northern Ethiopia where data are scarce.

## 2. Methods and Subjects

A cross-sectional study was conducted in type 2 diabetic patients attending outpatient diabetic clinic of Ayder Referral Hospital, Mekelle University, between December 2015 and April 2016 to evaluate the extent of diabetic target achievements and factors associated with them. All type 2 diabetic patients who were attending the outpatient diabetic clinic during the data collection period were included in the study provided that they met all of the following inclusion criteria: visited the diabetic clinic during the data collection period, had been diagnosed with diabetes at least six months earlier, had regular follow-up at diabetic clinic of Ayder Referral Hospital for at least 6 months prior to data collection, and agreed to sign consent form. During their regular follow-up, study participants were recruited into the study by trained data collectors. Patients were allowed to participate only once during the six-month period. Patients whose follow-up was not regular (those who missed three or more follow-ups within six months), patients with less than six-month follow-up, and newly diagnosed diabetic patients (less than six-month duration) were excluded from the study.

### 2.1. Data Collection

Data on sociodemographic variables (age, sex, income level, education, residency, and marital status), lifestyle variables (dietary variables, alcohol consumption, smoking, and physical activities), and self-monitoring and management practices were collected by trained diabetic nurses using face-to-face interviews and structured questionnaires. After face-to-face interview, information on anthropometric measurements, blood pressure, duration of diabetes, diabetic medications, coexisting comorbidities and other medications, lipid profiles, and current and previous five-month records of fasting plasma glucose (FPG) was collected through clinical laboratory investigation and review of patients' medical records.

### 2.2. Ethical Approval

Ethical clearance and approval for undertaking this study were obtained from Research and Community Service Council (RCSC) of Mekelle University, College of Health Sciences, before starting the actual data collection. Subsequent permission was obtained from the authorities of Ayder Referral Hospital, including medical director and the head of diabetic clinic. After explaining the aim and objectives of the study, verbal and written informed consent was obtained from each participant.

### 2.3. Measurements and Operational Definitions

We evaluated achievements of diabetes goal by assessing glycemic control, lipid profile, and blood pressure targets. We used mean and single FPG measurements for glycemic control and total cholesterol and triglyceride test for lipid profile assessments. Glycemic control was classified as good or poor based on the criteria of the American Diabetes Association (ADA) (good: FPG < 130 mg/dL; poor: FPG ≥ 130 mg/dL) [13].

Lipid profiles, blood pressure, and body mass index values were also categorized as normal and high based on criteria of ADA and US National Cholesterol Education Program. Accordingly, hypercholesterolemia was considered when total cholesterol level is ≥200 mg/dl and hypertriglyceridemia refers to a level ≥ 150 mg/dl. Dyslipidemia was defined as the presence of one or both of these abnormalities in serum. Hypertension was defined as systolic blood pressure ≥ 140 mmHg and/or diastolic blood pressure ≥ 90 mmHg or use of antihypertensive medications [13, 14].

Month to month variability of FPG was also assessed using mean, standard deviation (SD), and coefficient of variation (CV) [15]. Mean and standard deviations were calculated for each patient from six-month FPG data. Glycemic variability (GV) was then determined by dividing standard deviation to mean. GV was calculated only when more than three measurements were performed in the last six months including the final measurement done during data collection period. Wide GV was considered when CV of FPG was greater than 25% [15]. Similarly, ideal target for SD was considered when SD multiplied by three was less than mean [16]. We also calculated the approximate HbA1c from mean plasma glucose values using the following formula: mean plasma glucose (MPG) (mg/dl) = (35.6 *∗* HbA1c) − 77.3 [17].

### 2.4. Laboratory Analysis

Fasting serum value of FPG, total cholesterol, and triglyceride were determined according to their measurement principles and guidelines using Hitachi 902 Autoanalyzer (Roche Diagnostics, Germany). For total cholesterol determination, cholesterol esters in serum are hydrolyzed by cholesterol esterase in the reagent. The free cholesterol is then oxidized by cholesterol oxidase to the corresponding ketone liberating hydrogen peroxide, which is then converted to water and oxygen by the enzyme peroxidase. Para-aminophenazone (4-aminophenazone) takes up the oxygen and together with phenol forms a pink-colored quinoneimine dye and the absorbance was measured at 515 nm wavelength. Triglycerides were measured enzymatically in serum using a series of coupled reactions in which triglycerides were hydrolyzed to produce glycerol. Glycerol is then oxidized using glycerol oxidase, and H_2_O_2_, one of the reaction products, is measured quantitatively in a peroxidase-catalyzed reaction that produces a color. The color intensity is proportional to triglyceride concentration present in the sample. Absorbance is measured at 500 nm.

### 2.5. Data/Statistical Analysis

All data were entered into Epi Info software (version 7.1) and analyzed using Statistical Package for the Social Sciences (SPSS) software (version 20.0). First, data were cleaned, edited, and checked for completeness before analysis.

Chi-square test was used to measure the significant differences among different proportions. Binary logistic regression analysis was carried out to identify factors associated with poor glycemic control, hypertension, and dyslipidemia with their 95% confidence interval. At the same time, we performed covariate analysis between continuous variables. A *P* value of <0.05 was considered as a statistically significant level. All variables with a *P* value < 0.3 were considered for multivariate logistic regression to determine independent factors predicting poor diabetic control. Model fit to the data (validity and reliability) was assessed by the likelihood ratio *χ*^2^ test and Hosmer-Lemeshow goodness-of-fit test.

## 3. Result

### 3.1. Sociodemographic Characteristics of Study's Patients

From the total 188 participants, 104 (58.9%) were males. The median age of the study population was 54 years, ranging from 18 to 80 years. 34% of the study participants were illiterate and 17% were rural residents. Less than one-fifth (16.5%) of the participants have had regular physical exercises and 66% had food selection for their diabetes management. From participants with dietary restriction, 23.4% avoided sugar, salt, and fatty foods, while the rest used vegetables (13.8%), barley (8%), and teff (8%). Majority of the participants visited the diabetic clinic only 3-4 times in the last six months. Only 11.2% patients had their own glucometer for self-monitoring practices (Table 1). Table 1 summarizes the detailed sociodemographic and lifestyle features of the study's participants.

### 3.2. Clinical Characteristics

**Figure 1:**
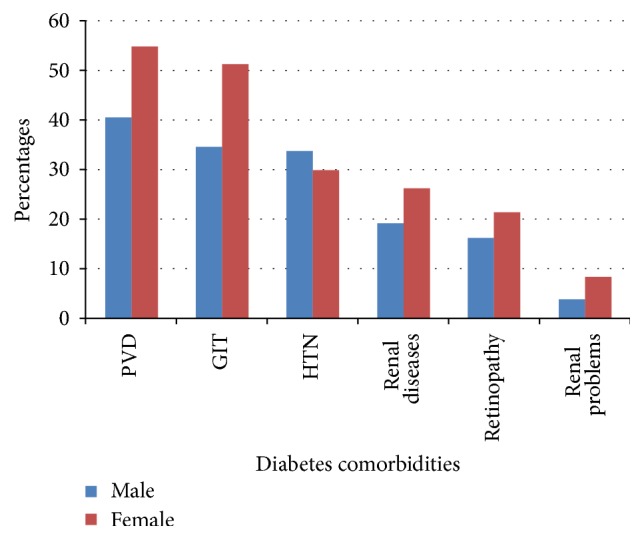
Prevalence of diabetes comorbidities among diabetic patients attending outpatient diabetic clinic of Ayder Referral Hospital. PVD: peripheral vascular diseases; GIT: gastrointestinal problem; HTN: hypertension.

Mean duration of diabetes since diagnosis was 6.5 years. A larger proportion of patients (55.3%) were taking oral hypoglycemic agents followed by insulin (37.8). In addition to diabetic treatments, about 40% of the patients were taking other medications. Based on self-report and clinical record review, comorbidities other than hyperglycemia were described in 68.1% of the patients. Peripheral vascular disease (46.8%), hypertension (44.7%), gastrointestinal problems (42.2%), renal diseases (22.3%), retinopathy (18.6%), and heart problems (5.9%) were among the reported comorbidities. Except hypertension, female proportions were higher in all comorbidities (Figure 1).

Based on coefficient of variation of FPG, 93 (49.5%) had marked month to month glycemic variations (CV: FPG ≥ 25) and 61 (32.44%) failed to achieve target SD for FPG (SD *∗* 3 < mean). Only 35 (18.6%) patients had HbA1c test record during the last six months. From these, 26/35 had HbA1c value ≥ 7% (poor control). The detailed clinical characteristics of the study's patients are indicated in Table 1.

### 3.3. Achievements of Diabetic Goals

Based on FPG measurements, 136 (72.3%) had poor glycemic control. After calculating HbA1c values from the corresponding mean FPG values, 110 (58.5%) had HbA1c value ≥ 7 (poor control), while 52.2% of patients were hypertensive and 58.5% had dyslipidemia and either hypercholesterolemia or triglyceridemia or both. Combined glycemic, lipid, and blood pressure targets were achieved only in 8.5% of the participants. While majorities (41.5%) achieved only one of these three goals, 22.3% did not achieve all the three targets. More proportion of males achieved combined targets than females (Table 2).

### 3.4. Factors Associated with Poor Diabetic Goals Achievements

Large standard deviation marked FPG variability and longer duration of diabetes were predictors of overall mean glycemic control. Patients with longer duration of diabetes were less likely to achieve overall mean glycemic targets. Similarly, patients with large month to month FPG variability and large standard deviation were more like to have high average FPG. All variables with *P* value less than 0.3 in binary logistic regression analysis were included in multivariate logistic regression analysis after checking for confounders. In multivariate analysis, these variables retained their significant association (Table 3). Higher body mass index (BMI ≥ 25) and presence of comorbidities other than hyperglycemia were variables that showed statistically significant association with hypertension. Female gender was also significantly associated with dyslipidemia (Table 3).

## 4. Discussion

This study revealed that only 8.5% of the study population achieved the combined glycemic, lipid, and blood pressure targets and 22.3% of the patients achieved none of these targets. While more than two-thirds of the patients had poor glycemic control, above half of the participants have had poor lipid and blood pressure control. A significant portion of the patients (68.1%) had also comorbidities other than hyperglycemia. Our result is lower as compared to the previous study in Israel, which found 13% of combined target achievements [18]. However, they used low-density lipoprotein cholesterol (LDL-cholesterol) for lipid profile and HbA1c for glycemic control as well as a lower cutoff point for blood pressure assessment, which is different from us. In line with our finding, similar rate of poor glycemic control was reported from Tanzania (69.7%) [19]. However, we found slightly higher prevalence of poor glycemic control based on mean FPG (72.3%) and lower results according to HbA1c calculated from mean FPG (58.5%). But we should not use the calculated HbA1c, since approximation results will mislead to wrong inferences. Several other studies described prevalence of poor glycemic control using HbA1c test results. Regardless of these parametric differences, relatively higher rate of poor glycemic control was reported from Malaysia (76%) [20], Saudi Arabia (73%) [21], and South Africa (74.6%) [22], while roughly similar results were found in Jordan (65.1%) [23] and Kuwait (66.7%) [24]. Lower prevalence rate of poor glycemic control was also reported in studies from Pakistan (46.7%) [25] and Spain (50.6%) [26]. Nevertheless, these countries, except Spain, are known for their highest prevalence of diabetes in the world.

In this study, we used FPG test as a tool for assessing glycemic control status. Despite the fact that assessment of FPG is insufficient to obtain optimal glycemic control [27–29] and to achieve long-term diabetic targets, markers such as HbA1c and postprandial plasma glucose tests are not available in the study area. On the other hand, lack of these tests could prevent patients and physicians from taking the necessary actions on time to improve and sustain glycemic control for long time. In fact, in this study, the proportion of patients with high mean FPG value was higher than the proportion of patients with high single FPG measurements (72.3% versus 67.0%). This may partly explain the high rate of poor diabetic goals achievements and high incidences of diabetes comorbidities in the study area.

For blood pressure targets, we found comparable results with studies from Israel (51%) [18] and South Africa (49.6%) [22] and lower than study reports from Brazil (63.2%) [30]. Most of the previous studies used LDL-cholesterol as a tool for assessing lipid profile targets. But because of inaccessibility of the LDL-cholesterol test, we used total cholesterol and triglycerides tests to assess lipid profile targets despite the fact that LDL-cholesterol is more associated with pathogenesis of cardiovascular diseases risks in type 2 diabetic patients.

In bivariate and multivariate analyses, longer duration of diabetes disease (AOR: 3.4) and wide month to month FPG variability as measured by large CV of FPG and large SD (AOR: 2.5) were significantly associated with overall poor mean FPG results. None of these variables were associated with single FPG values. Association of marked FPG variability (CV ≥ 25) with poor mean FPG control, but not with single FPG value, indicates that FPG variability mainly affects the overall long-term results. This can be interpreted as follows: diabetic patients who failed to narrow their month to month FPG variability and maintain its stability are less likely to achieve their long-term control. This may be in turn the reflection of low treatment adherence, low self-management practices, or irregular food intakes.

In accordance with our result, Assunção et al. and Juarez et al. also found significant association between longer duration of diabetes and poor glycemic control [30, 31]. This association may be due to the decreases in *β*-cell function and insulin secretion as the disease progresses over time.

Similarly, in bivariate analysis, patients with higher BMI (BMI ≥ 25), taking drugs other than antidiabetics, and having coexisting morbidities were more likely to be hypertensive and failed to achieve blood pressure targets. Female diabetic patients and patients with high FPG were also at high risk of developing dyslipidemia (*P* < 0.05). The association of high BMI with hypertension and female gender and high FPG with dyslipidemia is consistent with the pathogenesis and risk of these diseases. However, the association between presence of coexisting morbidities and hypertension is hard to explain, since frequencies were generated from the data collected by combination of self-report and review of clinical records.

According to clinical record review and self-reports, 68.1% of the study participants have had comorbidities other than hyperglycemia in which peripheral vascular diseases, gastrointestinal problems, hypertension, and renal diseases were most common. Particularly, the high rate of gastrointestinal problem is something uncommon and needs further investigation. Strong evidences demonstrated that poor glycemic control is associated with the development of diabetes complication and other cardiovascular effects [32–34]. However, diabetic patients with multiple disorders could also be challenged by the coexisting comorbidities to sustain their glycemic control and to attain target values.

As a limitation, this study was based on six months' retrospective data analysis and used only routine diagnostic tests available. Another prospective study with inclusion of other diagnostic markers such as postprandial plasma glucose, low-density lipoprotein, and HbA1c is required to fully investigate the prognostic value of glycemic variability and factors affecting it in resource-poor settings.

In conclusion, the results of this study showed that achievements of combined diabetic goals are very poor (only 8.5%). In order to improve combined diabetic goal attainment, patients should be advised to minimize month to month FPG variation and maintain consistent glycemic control.

## Figures and Tables

**Table 1:** Proportion of type 2 diabetic patients with poor glycemic control according to the sociodemographic, lifestyle, and clinical characteristics, attending Ayder Referral Hospital.

Patient characteristics	Total number (*N* = 188), *n* (%)	Mean glycemia ≥ 130 mg/dL (*N* = 136), *n* (%)	*P* value	Current glycemia ≥ 130 mg/dL *N* = 126, *n* (%)	*P* value
Age, mean + SD	49.9 + 16.9		0.36	49.9 + 16.9	0.68
≤30 years	5 (2.6)	3 (2.2)	0.7	1 (0.07)	0.04
31–45 years	43 (22.9)	33 (24.3)		29 (23.0)	
46–60 years	88 (46.8)	61 (44.9)		65 (51.6)	
>60 years	52 (27.7)	39 (28.7)		31 (24.6)	
Sex: male, *n* (%)	104 (58.9)	71 (52.2)	0.17	64 (50.8)	0.08
Education, *n* (%)					
Illiterate	64 (34.0)	49 (36.0)	0.28	47 (37.3)	0.09
Primary education	38 (20.2)	28 (20.5)		22 (17.5)	
Secondary and above	86 (45.7)	59 (43.3)		57 (45.2)	
Marital status, *n* (%),					
Single	36 (19.1)	27 (19.6)	0.51	24 (19.1)	0.85
Married	152 (80.8)	109 (80.4)		102 (80.9)	
Residency, *n* (%)					
Rural	32 (17.0)	26 (19.1)	0.25	23 (18.3)	0.6
Urban	156 (82.9)	110 (80.9)		103 (81.7)	
Income (ETB), *n* (%)					
None	34 (18.1%)	21 (15.4)	0.9	20 (14.7)	0.26
<1000	50 (26.6)	37 (27.2)		36 (28.6)	
1000–2500	56 (29.8)	44 (32.4)		42 (33.3)	
>2500	48 (25.5)	34 (25.0)		28 (22.2)	
Lifestyles, yes, *n* (%)					
Smoking	2 (15)	2 (1.5)	NA	2 (1.5)	
Alcohol intake	11 (5.9)	7 (5.1)	0.5	6 (4.8)	
Physical activity	31 (16.5)	21 (15.4)	0.47	17 (13.5)	0.11
Dietary restriction	124 (66.0)	86 (63.2)	0.31	79 (62.7)	0.28
Self-monitoring practices, yes, *n* (%)	21 (11.1)	15 (11.0)	0.64	12 (9.5)	0.84
Number of visits in 6 months					
3-4 times	106 (56.4)	75 (55.1)	0.58	76 (60.3)	0.12
5-6 times	82 (43.6)	61 (44.9)		50 (39.7)	
Duration of diabetes in years, mean + SD	6.5 + 6.8	-	**0.014**	6.5 + 6.8	0.21
<5 years	94 (50.0)	62 (45.6)	**0.007**	60 (47.6)	0.14
5–10 years	50 (26.6)	39 (28.7)		36 (28.6)	
>10 years	44 (23.4)	35 (25.7)		30 (23.8)	
Glycemic variability ≥ 25	93 (49.5)	77 (56.6)	**0.002**	62 (49.2)	0.91
Large SD (SD *∗* 3 > mean)	61 (32.44)	51 (37.5)	**0.02**	39 (31.0)	0.53
Diabetes drugs, *n* (%)					
OHA	104 (55.3)	73 (53.7)	0.67	68 (54.0)	0.14
Insulin only	71 (37.8)	54 (39.7)		46 (36.5)	
Combination therapy	13 (6.9)	9 (6.6)		12 (9.5)	
Use of other drugs, yes, *n* (%)	76 (40.4)	55 (40.4)	0.99	53 (42.1)	0.51
Presence of comorbidities, yes, *n* (%)	128 (68.1)	93 (68.4)	0.88	85 (67.5)	0.79
BMI ≥ 25	63 (33.5)	41 (30.1)	0.06	39 (31.0)	0.23
Dyslipidemia	110 (58.5)	85 (62.5)	0.10	80 (63.5)	**0.04**
Hypertension	98 (52.1)	70 (51.5)	0.59	65 (51.6)	0.7

SD: standard deviation; ETB: Ethiopian Birr; OHA; oral hypoglycemic agent, BMI: body mass index. *P* values were calculated using chi-square test.

**Table 2:** Achievements of diabetic goal in patients attending Ayder Referral Hospital, Northern Ethiopia.

Characteristics	Total number (%)	Female	Male	*P* value
FPG				
Normal (FPG < 130)	62 (33.0)	22 (26.2)	40 (38.5)	0.075
Poor control (FPG ≥ 130)	126 (67.0)	62 (73.8)	64 (61.5)	
Mean FPG				
Normal (FPG < 130)	52 (27.7)	19 (22.6)	33 (31.7)	0.17
Poor control (FPG ≥ 130)	136 (72.3)	65 (77.4)	71 (68.3)	
HbA1c (calculated)				
<7%	78 (41.5)	32 (38.1)	46 (44.2)	0.40
≥7%	110 (58.5)	52 (61.9)	58 (55.8)	
Hypertension				
No	84 (44.7)	43 (51.2)	41 (39.4)	0.093
Yes	98 (52.1)	38 (45.2)	60 (57.7)	
Dyslipidemia				
No	71 (37.8)	25 (29.8)	46 (44.2)	0.028
Yes	110 (58.5)	57 (67.9)	53 (51.0)	
Combined diabetic goal achievements^*∗*^				
All three goals achieved	16 (8.5)	6 (7.1)	10 (9.6)	0.43
Two goals achieved	52 (27.7)	19 (22.6)	33 (31.7)	
Only one goal achieved	78 (41.5)	39 (46.4)	39 (37.5)	
None of the goals achieved	42 (22.3)	20 (23.8)	22 (21.2)	

FPG: fasting plasma glucose; HbA1c: glycated hemoglobin. *P* values were calculated using binary logistic regression analysis. ^*∗*^Combined diabetic goal: combination of glycemic, lipid, and blood pressure targets.

**Table 3:** Bivariate and multivariate analysis for factors associated with wide mean glycemia, hypertension, and dyslipidemia in type 2 diabetic patients attending Ayder Referral Hospital, Northern Ethiopia (*N* = 188).

Characteristics	COR (95% CI)	*P* value	AOR (95% CI)	*P* value
*Variables associated with mean glycemia*				
Duration of diabetes				
<5 years	Reference			
5–10 years	2.6 (1.13–5.93)	0.025	2.6 (1.12–6.01)	0.027
>10 years	3.5 (1.34–9.06)	0.011	3.4 (1.3–9.0)	0.013
Target SD				
SD *∗* 3 < mean				
SD *∗* 3 > mean	2.5 (1.16–5.5)	0.019	2.5 (1.13–5.5)	0.023
FPG variability				
CV < 25				
CV ≥ 25	2.9 (1.5–5.8)	0.002	R^*∗*^	R^*∗*^
Yes	2.0 (1.04–3.7)	0.038		
*Variables associated with hypertension*				
BMI				
BMI < 25	Reference			
BMI ≥ 25	3.0 (1.54–5.69)	0.001	2.03 (0.88–4.71)	0.098
Presence of other comorbidities				
No	Reference			0.081
Yes	2.9 (1.53–5.76)	0.001	2.114 (0.91–4.9)	
*Variables associated with dyslipidemia *				
Sex				
Male	Reference			
Female	2.0 (1.071–3.66)	0.029	1.9 (1.002–3.48)	0.049
FPG				
FPG > 130	Reference			0.065
FPG ≥ 130	1.95 (1.04–3.67)	0.038	1.8 (0.96–3.46)	

COR: crude odd ratio; AOR: adjusted odd ratio; CI: confidence interval; SD: standard deviation; CV: coefficient of variation; BMI: body mass index; FPG: fasting plasma glucose. R^*∗*^ indicates that FPG variability was removed from multiple logistic regression analysis because of significant correlation with target SD.

## Abbreviations

ADA: American Diabetes Association
BMI: Body mass index
CV: Coefficient of variation
FPG: Fasting plasma glucose
GV: Glycemic variability
HbA1c: Glycosylated hemoglobin
IDF: International Diabetes Federation
SD: Standard deviation
RCSC: Research and Community Service Council.
